# Removal of Koos IV acoustic neuroma and auditory brainstem implant in NF2 patient

**DOI:** 10.3171/2021.7.FOCVID2188

**Published:** 2021-10-01

**Authors:** Maria Rosaria Scala, Pietro Spennato, Antonio Della Volpe, Claudia Santoro, Stefania Picariello, Alfonso Maria Varricchio, Claudio Ruggiero, Serena dé Santi, Giuseppe Cinalli

**Affiliations:** 1 Department of Pediatric Neurosurgery and; 2 Otology and Cochlear Implant Unit, Santobono-Pausilipon Children’s Hospital, Naples;; 3 Neurofibromatosis Referral Centre, Department of Woman, Child and General and Specialized Surgery, University of Campania “Luigi Vanvitelli,” Naples; and; 4 Departments of Physical and Mental Health, and Preventive Medicine, University of Campania “Luigi Vanvitelli,” Naples, Italy

**Keywords:** neurofibromatosis type 2, auditory brainstem implant, bilateral acoustic neuroma, meningioma, deafness

## Abstract

The authors present the case of removal of a Koos grade IV right acoustic neuroma in a neurofibromatosis type 2 (NF2) patient, already operated on for left cerebellopontine angle meningioma at 7 years of age and a left acoustic neuroma at 16 years of age. A transpetrosal approach allowed cochlear sensor implantation to detect residual hearing. An enlarged retrosigmoid approach then allowed subtotal microsurgical removal of the lesion; consequently, the authors illustrate the technical nuances of an auditory brainstem implant (ABI). One month after surgery, the ABI was successfully switched on, giving back hearing perception to the patient.

The video can be found here: https://stream.cadmore.media/r10.3171/2021.7.FOCVID2188

## Transcript

In this video we will present the removal of a Koos grade IV acoustic neuroma followed by auditory brainstem implant in an NF2 patient.

This 16-year-old girl affected by NF2 had already been operated in our department of complete removal of a left CP angle transitional meningioma. Here you can see the pre- and postoperative MRI with bilateral Koos grade I neuromas, followed by minor surgeries for CSF circulation troubles.

In 2020 she was operated on for subtotal removal of left acoustic neuroma, but in the following 2 years, despite bevacizumab therapy, regrowth of the left residual and rapid volume increase of the right neuroma with severe hearing loss was observed.^1^

### 1:04 Clinical Presentation

Neurological examination at admission showed profound hearing loss in the right ear and deafness in the left ear, gait instability, positive Romberg, left facial nerve palsy, and nystagmus in all directions.

Preoperative MRI showed a large right acoustic neuroma, significant regrowth of the left tumor, and several other small enhancing tumors compatible with the NF2.

Right retrosigmoid approach for tumor removal and auditory brainstem implant was decided. Retrosigmoid approach allows good microsurgical control of the whole tumor mass from the lower pole to the upper pole. For the intracanalicular component, removal of the posterior wall of the internal acoustic canal would be necessary with good control of the most hidden part of the tumor.

Left lateral position in 3-pins head frame under IOM and magnetic navigation. Incision was larger than standard retrosigmoid approach in order to allow partial mastoidectomy and cochleostomy and provide place for subcutaneous implant.

### 2:03 Mastoidectomy, Cochleostomy, and Test Cochlear Electrode Implant

Here the ENT team is performing the partial mastoidectomy in order to identify the cochlea, perform a cochleostomy, and implant a test cochlear electrode to detect residual hearing.^2^ In case residual hearing is present after neuroma resection, cochlear implant instead of brainstem implant could be indicated.^3,4^ Here we are implanting the cochlear electrode inside the cochleostomy.

### 2:32 Retrosigmoid Approach

After fixation of the cochlear electrode, we then start with a standard retrosigmoid approach. After the retraction of the cerebellum, we open the cistern in order to release CSF and facilitate further cerebellum retraction. Large opening of the arachnoid allows to expose the tumor very nicely, and mild retraction of the cerebellum allows significant exposure of the tumor. After coagulation of the outer part of the tumor, we can incise the outer capsula and perform biopsy and extensive surgical debulking with the ultrasonic aspirator. Significant debulking is necessary in order to allow further dissection of the tumor from the cerebellum. As you can see, it is not necessary to remove cerebellar parenchyma as proposed by some authors; it is always possible to perform slow and careful dissection of the tumor from the cerebellum, progress with ultrasonic aspirator and significant progressive debulking, and expose progressively the outer part of the tumor.

### 3:44 Dissection of the Upper Pole of the Tumor

Here in the upper part of the tumor volume, we are trying to identify the facial nerve in order to protect it during the further phases of the dissection, and here we can see the facial nerve and we can isolate it and continue with the debulking and removal of the tumor safely without risk to damage the facial nerve during these phases. This phase of identification is mandatory in order to try to save the facial nerve, although in such a large tumor and especially in an NF2 patient it is always very difficult to protect and respect the facial nerve that, as you can see, is extremely squeezed by tumor compression, but we try to keep the integrity of the facial nerve throughout the surgery, dissecting it slowly from the outer capsule of the acoustic neuroma. The bleeding is usually relatively easy to control even in the dissection of the outer capsule, and we can continue the dissection of the facial nerve throughout the circumference of the upper and posterior pole of the tumor.

### 5:15 Dissection of the Lower Pole of the Tumor

Then, we continue by isolation of the lower part of the tumor, trying to identify the lower cranial nerves and trying to identify the brainstem. Continue alternance of debulking and dissection is necessary until finally we can find and identify clearly the lower part of the tumor after the significant debulking that has been performed with the ultrasonic aspirator.

Here we can see, very nicely, the lower plan of dissection that allows visualization of the brainstem surface and lower cranial nerves. Here is the brainstem visible, and we continue then with further ultrasonic aspirator internal debulking. We identify the lower cranial nerves below the lower pole of the tumor, respecting them of course. And we continue the progressive dissection of the outer capsule from the lower part of the brainstem.

### 6:02 Opening the Internal Auditory Meatus

Then we continue with the complete isolation and identification of the adhesions between the tumor, and we can limit finally the residual tumor to the intracanalicular part and to the part that is bulging from the internal meatus inside the cistern. At this point, we decompress and we debulk the part that is bulging from the internal acoustic meatus. So we open the posterior wall of the internal acoustic meatus in order to expose the tumor part that is inside the internal acoustic meatus in order to try to perform removal as complete as possible in this phase.

### 6:56 Identification of the Foramen of Luschka

Due to the loss of any auditory response in the cochlear nerve, we performed the largest possible removal, without any attempt to preserve the residual eighth nerve fibers, and we decide for the auditory brainstem implant. Here the transducer is located under the skin and we look for the foramen of Luschka that is easily identified in the lower part of the surgical field and identifiable by the presence of the choroid plexus.

### 7:24 Insertion of the Electrode Inside the Foramen of Luschka

Then we take the electrode that it is inserted in the surgical field and we look for the best possible location for the electrode. The electrode itself is composed of 12 microelectrodes that must be placed in the closest possible contact with cochlear nuclei in the brainstem inside the Luschka foramen. Positioning of the electrode in close contact with the auditory nuclei of the brainstem may require some dissection of the foramen of Luschka to obtain the best possible anatomical contact between the implant pedal and the cochlear nuclei region. Here you can see the position of the 12 electrodes and of the 13th reference electrode that is in the middle of the electrode itself.

### 8:19 Adjustment of the Position of the Electrode Under Intraoperative Electrophysiological Monitoring

After first placement inside the foramen of Luschka we realize that the placement is not ideal because of the presence of the choroid plexus that does not allow the complete unfolding of the support of the electrode. Intraoperative stimulation shows that only four electrodes can have response, so the electrode must be repositioned, the shape of the Luschka foramen is adapted, and the orientation of the electrode needs to be modified. And we found that with a simple angle more upward directed allows the introduction of few millimeters more of the electrode well inside of the Luschka foramen. At this point, intraoperative monitoring shows that nine electrodes are in good position with a good response, so we leave the electrode as it is in this final position.

### 9:16 Outcomes

Postoperative MRI shows good placement of the electrode and nice removal of the tumor. Postoperative CT scan shows that the electrode is in good position inside of the foramen of Luschka. The patient presented postoperative facial cranial nerve palsy (House-Brackmann score IV). One month following the surgery, the auditory brainstem implant has been successfully switched on. The last audiogram, 2 months after surgery, disclosed already a huge improvement of the right hearing threshold levels (40 dB).

### 9:50 Conclusions

In conclusion, bilateral acoustic neuromas associated with NF2 remain one of the most challenging forms of acoustic neuromas. Bevacizumab can delay progression, but it is not a definitive solution. Surgery needs to be timely in the attempt to save residual hearing. In advanced cases, with profound hearing loss, auditory brainstem implant at the time of neuroma removal is a feasible option to restore some hearing capacity.^5,6^

## Disclosures

The authors report no conflict of interest concerning the materials or methods used in this study or the findings specified in this publication.

## Author Contributions

Primary surgeon: Cinalli. Assistant surgeon: Scala, Spennato, Della Volpe, Varricchio, Ruggiero, dé Santi. Editing and drafting the video and abstract: Cinalli, Scala, Picariello. Critically revising the work: Cinalli, Spennato, Santoro. Reviewed submitted version of the work: Cinalli, Santoro. Approved the final version of the work on behalf of all authors: Cinalli. Supervision: Cinalli.
